# Testing IgG antibodies against the RBD of SARS-CoV-2 is sufficient and necessary for COVID-19 diagnosis

**DOI:** 10.1371/journal.pone.0241164

**Published:** 2020-11-23

**Authors:** Victoria Indenbaum, Ravit Koren, Shiri Katz-Likvornik, Mayan Yitzchaki, Osnat Halpern, Gili Regev-Yochay, Carmit Cohen, Asaf Biber, Tali Feferman, Noy Cohen Saban, Roni Dhan, Tal Levin, Yael Gozlan, Merav Weil, Orna Mor, Michal Mandelboim, Danit Sofer, Ella Mendelson, Yaniv Lustig

**Affiliations:** 1 Central Virology Laboratory and Sheba Medical Center, Ministry of Health, Tel-Hashomer, Israel; 2 School of Public Health, Sackler Faculty of Medicine, Tel-Aviv University, Tel-Aviv, Israel; 3 Infection Prevention & Control Unit, Sheba Medical Center, Ramat-Gan, Israel; 4 Department of Immunology, Weizmann Institute of Science, Rehovot, Israel; Monash University, AUSTRALIA

## Abstract

The COVID-19 pandemic and the fast global spread of the disease resulted in unprecedented decline in world trade and travel. A critical priority is, therefore, to quickly develop serological diagnostic capacity and identify individuals with past exposure to SARS-CoV-2. In this study serum samples obtained from 309 persons infected by SARS-CoV-2 and 324 of healthy, uninfected individuals as well as serum from 7 COVID-19 patients with 4–7 samples each ranging between 1–92 days post first positive PCR were tested by an “in house” ELISA which detects IgM, IgA and IgG antibodies against the receptor binding domain (RBD) of SARS-CoV-2. Sensitivity of 47%, 80% and 88% and specificity of 100%, 98% and 98% in detection of IgM, IgA and IgG antibodies, respectively, were observed. IgG antibody levels against the RBD were demonstrated to be up regulated between 1–7 days after COVID-19 detection, earlier than both IgM and IgA antibodies. Study of the antibody kinetics of seven COVID 19 patients revealed that while IgG levels are high and maintained for at least 3 months, IgM and IgA levels decline after a 35–50 days following infection. Altogether, these results highlight the usefulness of the RBD based ELISA, which is both easy and cheap to prepare, to identify COVID-19 patients even at the acute phase. Most importantly our results demonstrate that measuring IgG levels alone is both sufficient and necessary to diagnose past exposure to SARS-CoV-2.

## Introduction

SARS-CoV-2 emerged in Wuhan, China during December 2019 [1] and since then has spread around the globe [2]. As of June 16 2020 there have been over 8 million COVID-19 cases and 430,000 deaths (https://coronavirus.jhu.edu/map.html).

The symptoms of SARS-CoV-2 infection vary widely, from asymptomatic disease to multisystem organ failure [3, 4]. Diagnosis of COVID-19 infection depends primarily on identification of SARS-CoV-2 RNA in nasopharyngeal samples using quantitative reverse transcriptase PCR (qRT-PCR) [5]. The qRT-PCR is considered both sensitive and specific, however, can only detect SARS-CoV-2 RNA during the acute phase of the disease. Moreover, as social distancing and border closures are slowly being released, identification of individuals who recovered from COVID-19 infection and have antibodies against the virus is essential.

In recent weeks several serological COVID-19 diagnostic kits have been commercially introduced which are based on detecting antibodies against all or part of either the Nuclecapsid (N) or the Spike (S) proteins of SARS-CoV-2 [6]. Most of these assays are aimed at detecting IgG antibodies as these are believed to be sufficient to confer protection against SARS-CoV-2 infection both individually and for the general public (herd immunity) [7]. Only limited data and in small cohorts is available regarding the performance of these assays and even more scarce data exist on the presence of other antibody types, such as IgM and IgA following COVID-19 infection.

Recently a detailed protocol for expression of antigens derived from the Receptor Binding Domain (RBD) of the S protein of SARS‐CoV‐2 that can serve as a substrate for immunological assays has been published by a group from Mount Sinai hospital [8, 9]. We have used this antigen to build three enzyme‐linked immunosorbent assays (ELISAs) that can detect IgM, IgG and IgA antibodies against SARS-CoV-2 and demonstrate here the performance of these assays using a large cohort of clinical samples from qRT-PCR positive COVID-19 patients and from individuals with no prior exposure to COVID-19.

## Materials and methods

### Patients

COVID-19 patients: The 309 serum samples included in the validation study and 41 serum samples included in the antibody kinetic study were obtained from 309 and seven persons, respectively, diagnosed by positive nasopharyngeal swab samples for SARS-CoV-2 RNA by qRT-PCR [5]. Only laboratory-confirmed cases were included. Serum samples were sub-divided into different categories based on the time (days) after the first positive COVID-19 qRT-PCR result (FPP): 1–7, 8–14, >14.

Sera from non-COVID-19 individuals: Three hundred and twenty-four serum samples from the Israeli Central Virology Laboratory (iCVL) archives which were collected prior to September 2019 from individuals requesting polio immunization status were included as non-COVID-19 controls. All non-COVID-19 individuals were healthy at the time of collection. No antibodies against potential SARS-CoV-2 cross-reactive pathogens were tested for the non-COVID-19 individuals, however, seasonal Human Coronavirus (HCoVs) infections is prevalent in Israel with more than 10% of Influenza-like illness molecularly diagnosed annually as HCoVs [10]. Furthermore antibodies against HCoVs are widespread in human sera [11]. Therefore cross-reactivity with HCoVs antibodies should be recognized by evaluating antibodies against SARS-CoV-2 in the Israeli non-COVID-19 population tested in this study.

### Ethics

The institutional review board of Sheba Medical Center approved the study and waived the requirement for informed consent because the data were analyzed anonymously. Approval from the Ethical Committee was granted before starting the study.

### Antigen preparation

The RBD antigen was prepared as described [8]. Briefly, The RBD sequence is based on the genomic sequence of the first virus isolated Wuhan-Hu-1 and optimized for mammalian cell expression. The natural Spike N terminal signal peptide (amino acids 1–14) was fused to the RBD amino acids 319–541 and also fused with a C-terminal hexahistidine tag to allow isolation. The recombinant protein was produced in Expi293F cells (Thermo fisher) by transfecting these cells with purified DNA using Expifectamine 293 kit (Thermo fisher). Three days post-transfection the cells were harvested and the RBD protein was isolated from the supernatant by 2 hours incubation at RT with HisPure Ni-NTA resin (Thermo). The RBD was eluted and dialyzed against PBS.

### ELISA

ELISA was first developed by evaluating the 450nm OD values of 12 negative and 4 positive COVID-19 samples using different concentrations of RBD antigen coating and HRP-conjugated secondary antibody for each of the 3 antibody isotypes (IgG, IgA and IgM). Optimal antigen amount per well and antibody concentrations were determined based on highest ratio of mean positive/ mean negative samples. Cut-off values for a positive result were set as the mean +3 SD of negative control sera (n  =  100). ELISA index value was defined as the ratio between sample and cut-off ODs. Samples were labeled borderline within ± 10% of the ELISA index value.

For the ELISA, a 96 well microtiter Polysorb plate (Nunc, Thermo, Denmark) was coated overnight at 4°C with 50μl per well of 1μg/ml of RBD antigen for detection of IgG and IgM antibodies and 2μg/ml for detection of IgA antibodies. After blocking with 5% skimmed milk at 25°C for 60 minutes, positive and negative control and human serum samples without replications and calibrator (sample at the cut-off O.D value) in triplicate (all diluted 1:100 with 3% skimmed milk), were added to antigen coated wells. The plate was incubated at 25°C for 120 minutes, washed and a HRP-conjugated isotype specific antibody (goat anti-human IgG horseradish peroxidase (HRP) conjugate (Jackson ImmunoResearch, PA, USA Code: 109-035-088) (diluted 1:15000), anti-human IgA HRP conjugate (Abcam, MA, USA, product number: ab7383) (diluted 1:2000) or goat anti-human IgM HRP conjugate (Jackson ImmunoResearch, PA, USA Code: 109-035-129) (diluted 1:20000)) was added to each well for 60 min. After washing, incubation of TMB Substrate Solution (Abcam) for 5 min and the addition of stop solution (2N HCl), the OD of each well was measured at 450nm using a micro-plate reader (Sunrise, Tecan). ELISA index value below 0.9 was considered negative, between 0.9 and 1.1, borderline and equal or above 1.1, positive.

### Statistical analysis

Sensitivity was defined as the proportion of patients identified as having current or previous SARS-CoV-2 infections among those who were initially diagnosed by real-time PCR in respiratory samples. Specificity was defined as the proportion of individuals who are or were not infected with SARS-CoV-2 using archive samples collected prior to September 2019. Primary sensitivity and specificity data were calculated with borderline results excluded, included as positives, and included as negatives (Table 2). However for subsequent analysis, performance was calculated with borderline results excluded because no repeat samples were available for retesting. The data are presented with 95% confidence interval (CI). Scatter plot of experimental groups was performed using GraphPad Prism 5.0 (GraphPad Software, Inc., San Diego, CA), by two-tailed parametric t-test means with confidence interval (CI) of 95%. BioVenn diagram was calculated and built as described [12].

## Results

Between March 3^rd^ and May 11^th^ 2020, 309 serum samples were collected from patients with acute or past confirmed COVID-19 infection. The demographic and clinical characteristics of the patients are shown in Table 1. The mean (±SD) age of the patients was 40 years (range, 13–85); 65% were men. Samples from the majority of patients (56%) were collected at least 29 days after first positive PCR. Most (94%) had mild symptoms while 20 (6%) patients were considered severe.

**Table 1:** Covid-19 patient’s characteristics.

	SARS-CoV-2 (n = 309)
**Age group**	Mean 40 (Range 13–85)
**Gender**	
Male (%)	65% (200/309)
Female (%)	35% (109/309)
**SARS-CoV-2 PCR Positivity**	100% (309/309 tested)
**Days Post SARS-CoV-2 Positive PCR**	
≤ 7 d	4% (11/309)
8–14 d	9% (28/309)
15–21 d	16% (50/309)
22–28 d	15% (46/309)
≥ 29 d	56% (174/309)
**Hospitalized**^**1**^	6% (20/309)

^1^Severe and critical disease patients.

**Fig 1:**
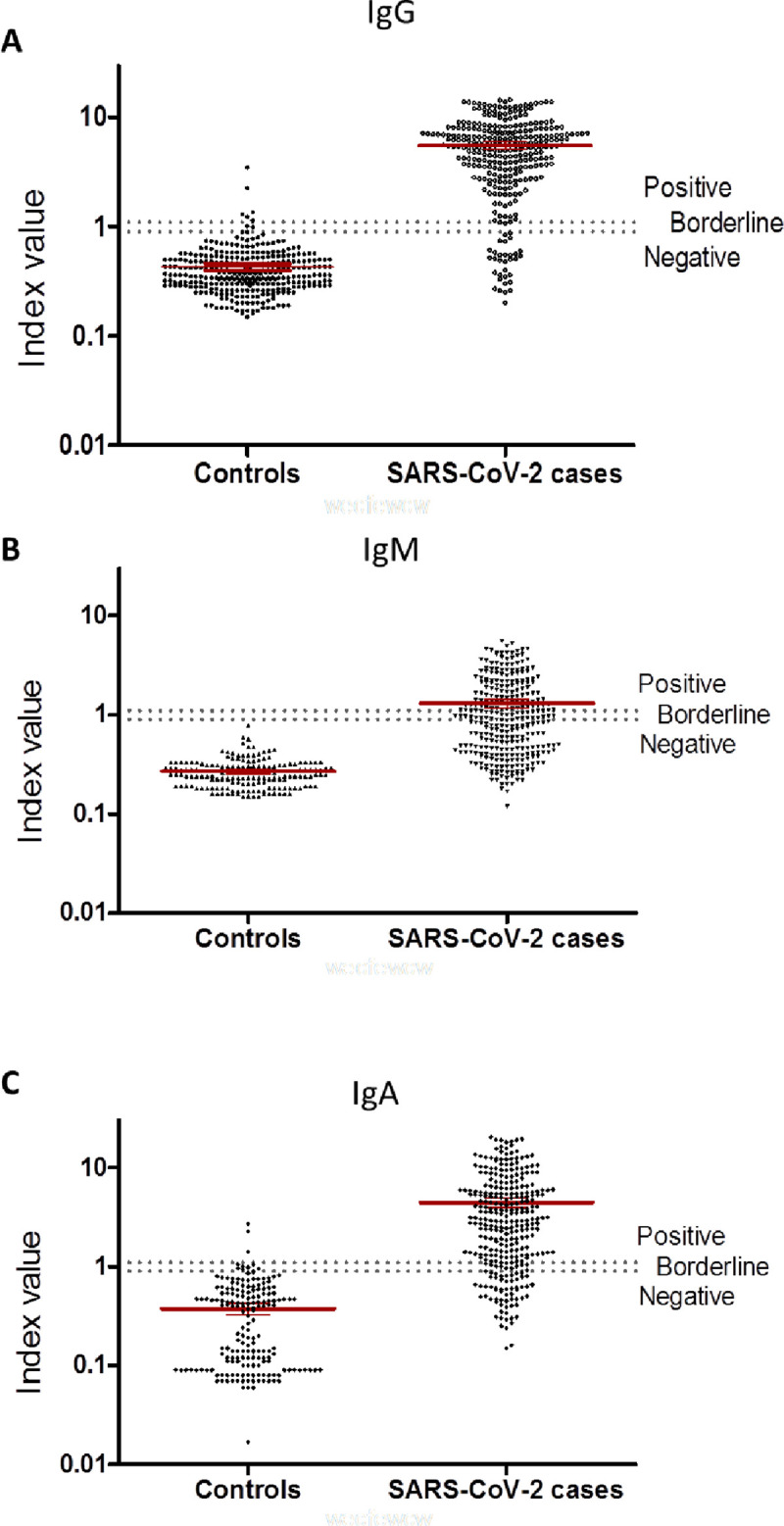
Scatter plots representing the distribution obtained for control and SARS-CoV-2 case sera for three ELISA’s. Red lines represent mean values with 95% confidence interval (CI). Dotted lines indicate the respective cut-off values to determine positive, borderline and negative test results. A) SARS-CoV-2 IgG antibody performance; B) SARS-CoV-2 IgM antibody performance; C) SARS-CoV-2 IgA antibody performance.

Scatter plot data of the 3 ELISAs (Fig 1) demonstrate that in all tests the cut-off can distinguish between negative and positive cases efficiently with significant differences between SARS-CoV-2 positive and negative samples (p<0.0001 for IgA, IgM and IgG). The specificities of the tests are very high and range between 98%-100% when borderline data is not included (Table 2A). Most interestingly sensitivity analysis shows that even when taking into account all the samples (including days 1–14 after first positive PCR), the highest sensitivity is achieved for detecting IgG antibodies (88%) while IgA is less sensitive with 80% and IgM with only 47% (Table 2A). Positive and negative predictive values (PPV and NPV) of 97% and 90% respectively are highest for the ELISA IgG (Table 2B).

**Table 2:** SARS-CoV-2 antibody performance using in house ELISA assay.

A. Clinical sensitivities and specificities			
		SARS-CoV-2 samples testing positive (sensitivity; 95% CI)	Control samples testing negative (specificity; 95% CI)
RBD IgG				
	Borderline data excluded from analysis	271/307 (88%; 84.7–91.9)	318/324 (98%; 96.7–99.6)
	Borderline data considered positive	273/309 (88%; 84.8–91.9)	318/326 (98%; 95.9–99.2)
	Borderline data considered negative	271/309 (88%; 84.0–91.4)	320/326 (98%; 96.7–99.6)
RBD IgM			
	Borderline data excluded from analysis	133/281 (47%; 41.5–53.2)	180/180 (100%)
	Borderline data considered positive	161/309 (52%; 46.5–57.7)	180/180(100%)
	Borderline data considered negative	133/309 (43%; 37.5–48.6)	180/180 (100%)
RBD IgA			
	Borderline data excluded from analysis	237/296 (80%;75.5–84.6)	170/173 (98%; 96.3–100)
	Borderline data considered positive	250/309 (81%; 76.5–85.3)	170/180 (94%; 91.1–97.8)
	Borderline data considered negative	237/309 (77%; 72.0–81.4)	177/180 (98%; 96.5–100)
B. Predictive values for SARS-CoV-2 antibody detection			
		PPV (%, 95% CI)	NPV (%, 95% CI)
	RBD IgG^2^	271/277 (98%, 96.1–99.5)	318/354 (90%; 86.7–93)
	RBD IgM^2^	133/133 (100%)	180/328 (55%; 49.5–0.6)
	RBD IgA^2^	237/240 (99%; 97.3–100)	170/229 (74.2%; 68.6–79.9)

^2^ Borderline data were discarded.

**Fig 2:**
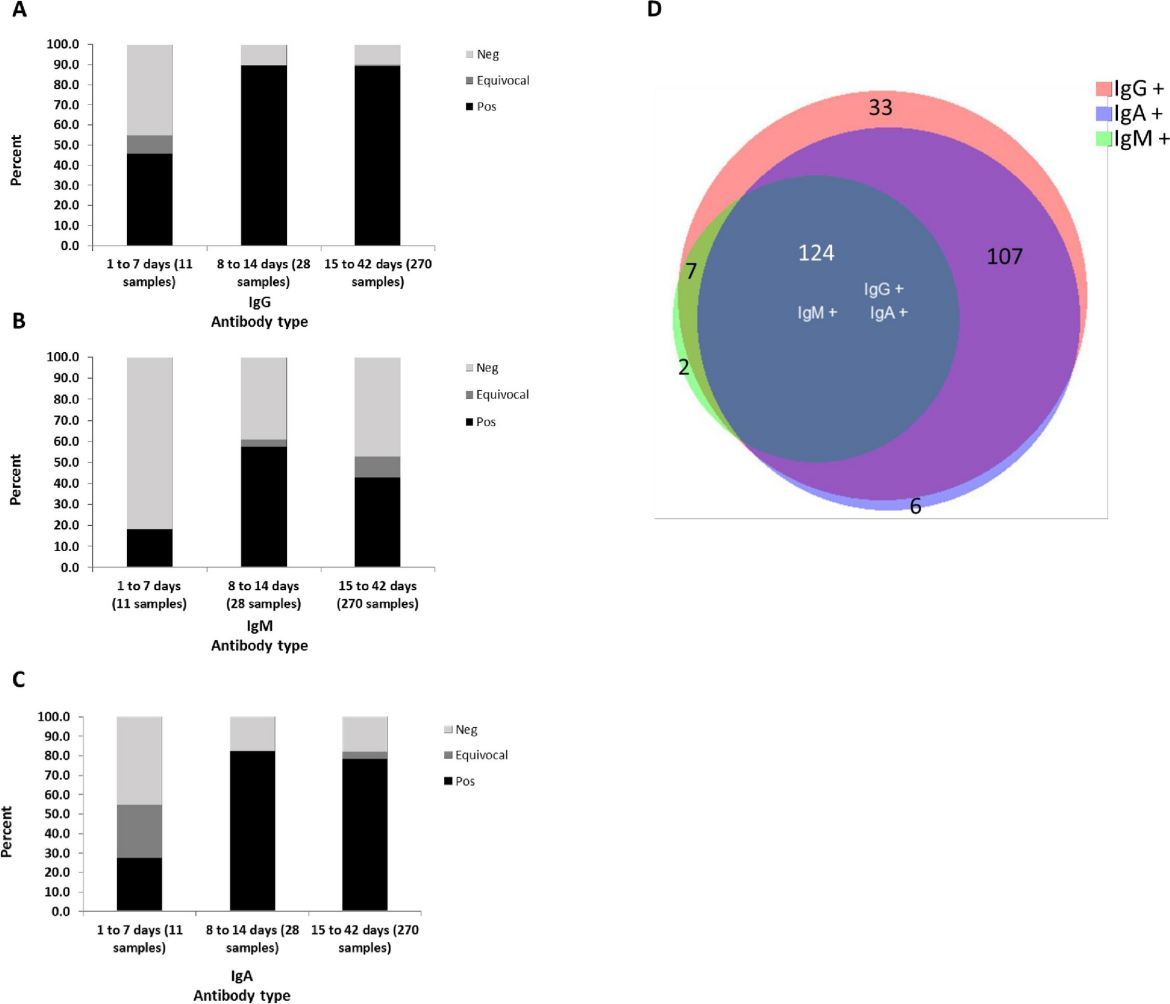
Sensitivity of RBD ELISA based on time from first positive PCR. Sensitivities of IgG (A), IgM (B) and IgA (C) in relation to the time passed since first positive PCR: 1 to 7 days (n = 11), 8 to 14 days (n = 28), and 15–42 days (n = 270). D. A BioVenn diagram resulting from comparison of positive results to IgG (orange), IgM (Green) and IgA (Blue) antibodies from 279 samples obtained from SARS-CoV-2 positive individuals which were identified by at least one of the immunoassays.

Sensitivity data based on time after FPP for the 3 antibody types are presented in Fig 2 and demonstrate that for COVID-19 positive samples collected less than 7 days after FPP- 18%, 26% and 44% of IgM, IgA and IgG antibodies, respectively, are detected by the ELISA. Most importantly, The ELISA detects IgM antibodies in 59% and 46% of samples collected 8–14 days and 15 and above days after FPP, respectively, and IgA and IgG antibodies in 81–82% and 89% of samples collected more than 8 days FPP, respectively. Comparison of positive results to IgG, IgM and IgA antibodies demonstrated that from 279 SARS-CoV-2 positive samples which were identified by at least one of the immunoassays 271 (97%), 133 (48%) and 237 (85%) of samples from SARS-CoV-2 positive individuals were identified by IgG-, IgM- and IgA-, RBD-based ELISA, respectively. Only 6 and 2 samples had IgA and IgM antibodies, respectively, without IgG antibodies (Fig 2D). No significant difference in assay sensitivity was observed when data was stratified by gender and days after FPP (S1 Table). Altogether, these results suggest that IgG antibodies are the most sensitive among all other antibody types and are produced very early after disease onset.

**Fig 3:**
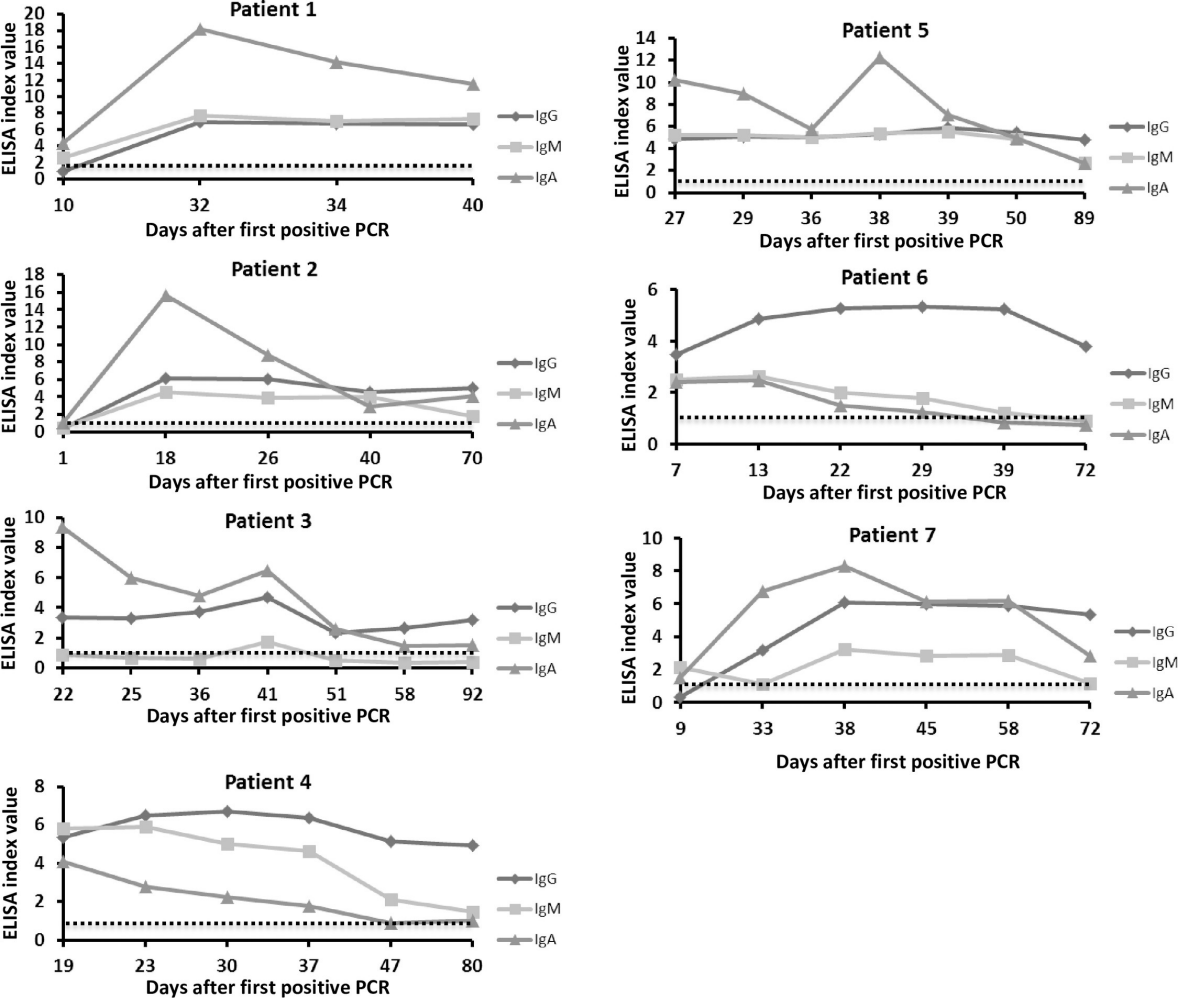
COVID-19 antibody kinetics. Four to seven serum samples were obtained from each of 7 COVID-19 patients (marked patients 1–7) and IgM, IgG and IgA antibody levels were measured. Dotted line marks index value of 1.1 which separates between levels considered positive (≥1.1) and borderline or negative (<1.1).

Antibody kinetics of IgM, IgA and IgG levels were evaluated on 7 COVID-19 patients with 4 to 7 samples each, obtained between 1–92 days after FPP. Fig 3 demonstrates that all 3 antibody types are induced between 1 and 10 days after FPP in most COVID-19 patients. Interestingly, high IgG levels are maintained for at least 3 months in all 7 cases while IgM and IgA levels decline after 35–50 days.

## Discussion

The COVID-19 pandemic has spread rapidly and in less than 3 months brought this uncharacterized disease to almost every part of the world. In an effort to quickly develop diagnostic tools, research laboratories worldwide have started sharing information about the SARS-CoV-2 virus. Since the RBD is a major target for human antibodies and is poorly conserved with other HCoVs, several research groups have constructed the RBD antigen and used it for identifying SARS-CoV-2 specific antibodies from COVID-19 positive and negative patients [8, 11]. Based on the published protocol for RBD construction [9], we developed an ELISA to detect IgM, IgA and IgG antibodies against COVID-19 and tested its performance in the Israeli population with hundreds of samples obtained from COVID-19 patients and non-COVID-19 individuals. This RBD ELISA is easy to prepare and the cost involved is primarily due to purchasing HRP conjugated secondary antibody, making this test available to many labs including in low income countries.

The results from this study demonstrate that IgG antibodies alone could be used to diagnose COVID-19 infection as they are detected even during the acute phase and can identify 97% of SARS-CoV-2 positive patients with a RBD-ELISA positive result. Our preliminary unpublished results show that detection of total antibody did not significantly increase the sensitivity compared to IgG alone. While a RBD based ELISA demonstrated high sensitivity for total antibody in a recent study [11], comparison of commercial antibodies detecting IgG or total antibodies against SARS-CoV-2 did not show significant differences [13]. Our results also show that IgM antibodies, traditionally known to be produced in the body following viral infection earlier than IgG [14], are significantly less detected than IgG antibodies even in early days post first positive PCR. Indeed two recent studies have shown that seroconversion for IgG and IgM against the RBD occurred simultaneously or sequentially [15, 16] while another study demonstrated that the IgA response is higher than IgM [17]. Altogether, our results suggest that detection of IgG antibodies alone, at least against the RBD, is sufficient but necessary for COVID-19 serological diagnosis in SARS-CoV-2 RNA positive patients.

Due to the widespread COVID-19 infection and the severe implications for public health, numerous COVID-19 serological diagnostic tools were developed and manufactured. As a consequence several studies have recently been published regarding the performance of these tests [18–21]. Interestingly there is a large variability in diagnostic test performance, especially sensitivity, between different assays and even differences in studies evaluating the same assay or the same antigen [22]. Using the RBD antigen as bait Premkumar et al. demonstrated sensitivities of 94%, 77.5% and 69% for IgG, IgA and IgM, respectively, in a cohort of 49 samples obtained from COVID-19 patients >9 days after onset of illness [11]. Other studies showed COVID-19 IgG and IgM sensitivities of 91% for 23 samples taken 19–42 days after symptoms [23] and IgG, IgA and IgM sensitivities of 96%, 92% and 98% respectively, for 53 samples >10 days post first positive COVID-19 PCR [24]. One possibility for the variability between all these studies including ours are differences in COVID-19 diagnosis between countries. It is also plausible that low number of samples evaluated in each of these previously published studies result in large confidence intervals that make these differences not significant. In addition, the severity of COVID-19 patients whose samples have been evaluated may also vary. Similarly to the clinical distribution of COVID-19 patients, our study was composed of samples obtained from 309 acute and recovered COVID-19 patients with mostly mild disease. Indeed several recent studies demonstrated that mildly ill patients have lower IgM and IgG responses against SARS-CoV-2 than severely ill patients [25–27] which can be the reason for the lower sensitivity observed in our cohort, especially regarding IgM.

This is one of the largest diagnostic evaluations that has been performed so far and as such can provide solid data for the presence of all 3 antibody types against the RBD. Due to the high specificity of this RBD ELISA, the sensitivities observed in this study are most probably an underestimation. It is possible that a small percentage of COVID-19 positive cases do not develop antibodies [28] and therefore the IgG, IgM and IgA false negative rates of 11%, ~50% and 19%, respectively detected here is lower.

A limitation of this study is that it only includes COVID-19 patients from Israel. One of the most interesting phenomenon emerging for COVID-19 global pandemic is the substantial variability between countries in both COVID-19 morbidity and mortality (https://ourworldindata.org/mortality-risk-covid). An open epidemiological question is whether there is a difference in the generation of antibodies against SARS-CoV-2 infection in individuals from different countries or different ethnicities and if this can be attributed to the mortality and morbidity rate. In addition, despite testing of COVID-19 patients with both mild and severe symptoms, asymptomatic SARS-CoV-2 PCR positive individuals were not assessed in this study. Therefore, future studies should investigate antibody kinetics against COVID-19 after asymptomatic infections.

In conclusion, due to the global emergence of SARS-CoV-2, there is significant importance in the establishment of serological diagnostic tools to evaluate the prevalence of COVID-19 in the population and detect individuals which were exposed to the virus. This study demonstrate that both IgA and IgG antibodies against the SARS-CoV-2 RBD can be used to detect COVID-19 patients even at the acute phase with high specificities and sensitivities while IgM sensitivity levels against the RBD are lower.

## Supporting information

S1 Table SARS-CoV-2 antibody performance stratified by gender and days after first positive PCR. (DOCX)
