# Supplementary material for: Testing IgG antibodies against the RBD of SARS-CoV-2 is sufficient and necessary for COVID-19 diagnosis
Source: PLoS One. 2020 Nov 23;15(11):e0241164. doi: 10.1371/journal.pone.0241164 (PMC7682882; doi:10.1371/journal.pone.0241164)
Supplement: S1 Table — (DOCX) [file pone.0241164.s001.docx]

|  | | Male  (sensitivity; 95% CI) | Female  (sensitivity; 95% CI) |
| --- | --- | --- | --- |
| RBD IgG | |  | |
| Days post PCR | 1-7 days | 4/8  (50%; 21.5-78.5) | 1/2  (50%; 9.5-90.5) |
|  | 8-14 days | 12/15  (80%; 54.8-92.9) | 13/13  (100%; 77.2-100) |
|  | 15-42 days | 155/175  (89%; 83-92.5) | 86/94  (91%; 84.1-95.6) |
| RBD IgM | |  |  |
| Days post PCR | 1-7 days | 1/9  (11%; 2-43.5) | 1/2  (50%; 9.5-90.5) |
|  | 8-14 days | 8/15  (53%; 30.1-75.2) | 8/12  (67%; 39.1-86.2) |
|  | 15-42 days | 77/156  (49%; 41.6-57.1) | 38/87  (44%; 33.7—54.2) |
| RBD IgA | |  |  |
| Days post PCR | 1-7 days | 2/6  (33%;9.7-70) | 1/2  (50%; 9.5-90.5) |
|  | 8-14 days | 12/15  (80%; 54.8-92.9) | 11/13  (85%; 57.8-95.7) |
|  | 15-42 days | 136/170  (80%; 73.3-85.3) | 75/90  (83%; 74.3-89.6) |

S1 Table: SARS-CoV-2 antibody performance stratified by gender and days after first positive PCR.
